# Haematological abnormalities as diagnostic indicators of malaria in returning travellers: a retrospective study at Mohamed V Military Instruction Hospital

**DOI:** 10.1099/acmi.0.000919.v3

**Published:** 2025-05-21

**Authors:** Nabil Mohamed Zniber, Hamid Laatiris, Hamza Siyar, Abdelouahab Erraji, Ismail Labrouzi, Mohamed Jnah, Mehdi Talbi, Maryem Iken, Badreddine Lmimouni, Hafida Naoui

**Affiliations:** 1 Department of Parasitology and Mycology, Mohamed V Military Instruction Hospital, Rabat, Morocco; 2 Faculty of Medicine and Pharmacy, Mohammed V University of Rabat, Rabat, Morocco

**Keywords:** diagnostic indicators, malaria, haematological abnormalities, thrombocytopenia, lymphocytopenia, returning travellers

## Abstract

**Introduction.** Malaria remains a significant global health concern, particularly in travellers returning from endemic regions. Haematological abnormalities are often associated with malaria and can serve as diagnostic indicators, especially when clinical symptoms are nonspecific.

**Objective.** This study aims to identify the most relevant haematological parameters for diagnosing malaria in travellers returning from endemic areas, who sought care at the Mohamed V Military Instruction Hospital in Rabat.

**Methods.** We conducted a retrospective comparative study involving 829 patients who returned from malaria-endemic regions between January 2017 and December 2023. Data collected included demographic information, parasitological test results and comprehensive haematological profiles. Statistical analysis was performed to determine the sensitivity and specificity of various haematological parameters in diagnosing malaria.

**Results.** Thrombocytopenia, lymphocytopenia and anaemia were the most significant haematological abnormalities associated with malaria. Thrombocytopenia, defined as a platelet count below 150×10³ µl^−1^, demonstrated a sensitivity of 75.91% and a specificity of 84.11%. Lymphocytopenia, with a threshold of less than 1.5×10³ µl^−1^, showed a sensitivity of 69.47% and a specificity of 78.39%. Anaemia, defined by haemoglobin levels below 13 g dl^−1^ in men and 12 g dl^−1^ in women, also significantly correlated with malaria diagnosis.

**Discussion.** This study highlights the significance of haematological abnormalities as key diagnostic markers for imported malaria cases. By analysing retrospective data, we observed that these abnormalities, especially thrombocytopenia and anaemia, are common amongst returning travellers with confirmed malaria. These findings suggest that clinicians can use such markers as a valuable tool for early malaria diagnosis, potentially improving patient outcomes. Additionally, the study reinforces the need for heightened awareness amongst healthcare providers in non-endemic regions regarding the presentation of malaria in travellers.

**Conclusion.** Haematological parameters such as thrombocytopenia, lymphocytopenia and anaemia are valuable diagnostic tools for malaria in returning travellers. These findings suggest that these parameters should be integrated into diagnostic protocols to improve the accuracy and timeliness of malaria diagnosis, particularly in clinical settings with limited access to advanced diagnostic tools.

## Data Summary

The dataset supporting the findings of this study has been provided in a supplementary table with the online version of the article. All data were anonymized to protect patient confidentiality and consist of haematological and parasitological results from 829 patients who returned from malaria-endemic regions between 2017 and 2023. The dataset includes blood test results (complete blood counts, platelet counts, etc.) and malaria diagnoses.

## Introduction

Malaria is a potentially deadly disease caused by protozoan parasites of the genus *Plasmodium*. This menace has continued to constitute a global threat, with over 200 million cases and 93,000 deaths reported in 2022 [1]. Most of these cases are linked to *Plasmodium falciparum* and are in Africa. Five species of *Plasmodium* are now recognized as causing human malaria, namely, *P. falciparum*, *Plasmodium vivax*, *Plasmodium ovale*, *Plasmodium malariae* and *Plasmodium knowlesi*; all five continue to pose significant public health threats. Infections may develop into multiple complications that usually lead to death.

Febrile illness is widespread in tropical countries. However, this sign is nonspecific for malaria, which makes fever one of the most sensitive signs whilst, simultaneously, posing a diagnostic challenge and ultimately delaying the necessary antimalarial treatment [2]. *Plasmodium* parasites are red blood cell parasites that frequently play a central role in the pathophysiology of malaria through numerous haematological parameter modifications. Amongst other parameters are platelets, lymphocytes, leucocytes and haemoglobin (Hb) levels [3].

Diagnosis with the sole help of haematology can be highly relevant for precisely diagnosing malaria in travellers from endemic countries. This study aims to determine the most specific and sensitive parameters related to malaria diagnosis [4].

## Methods

Our study consists of a retrospective comparative study outlining haematological abnormalities in patients with malaria returning from endemic areas and consulting at the Mohamed V Military Instruction Hospital in Rabat from January 2017 to December 2023. Data collected included demographic information, parasitological test results and haematological test results.

### Blood collection for malaria testing

Patients presenting to the emergency department suspected of having imported malaria underwent venous blood sampling via antecubital vein puncture into an EDTA tube (Vacuette). The tube was then sent to the Parasitology-Mycology department, where an on-call resident conducted the reading under the guidance of the responsible biologist.

### Malaria detection in blood

The sample was initially processed by preparing thick blood smears.

A drop of fresh blood was placed on a slide, defibrinated using a circular motion with the edge of a second slide and then dried in an incubator at 37 °C. After drying, the drop was haemolyzed in a 2% saponin solution. Once transparent, it was stained using the RAL Diagnostics S555 staining kit, starting with Fix RAL for 1 min and 30 s. This step allows for malaria diagnosis without species distinction.

For a thin blood smear, a drop of blood was placed on a slide and spread with a second slide inclined at 45°, forming a ‘cat tongue’ smear. Without a haemolysis step, it was stained directly with the same kit. This smear allows for species identification, staging and counting parasitized red blood cell proportions.

In parallel with the parasitological study, an EDTA tube containing the patient’s blood was sent to the haematology and immunohaematology laboratory for a complete blood count using the Beckman Coulter analyzer. The results include Hb level, platelet count (PLT), leucocyte count and differential leucocyte count, including neutrophil count (NEU), lymphocyte count (LYM) and monocyte count (MON).

### Statistical analysis

Statistical analysis was performed via Jamovi 2.4.11. Firstly, the normality of the data distribution was tested using the Shapiro–Wilk test. Continuous variables not conforming to a normal distribution were analysed using the Kruskal–Wallis test. Categorical variables were analysed using the chi-square test. Spearman’s correlation was used to test associations for non-normally distributed variables. Subsequently, logistic regression was used to predict malaria diagnosis utilizing the most discriminatory parameters. Moreover, the receiver operating characteristic (ROC) curve finally assessed the performance of our regression model.

## Results

During the study period, we collected complete data from 829 patients. The mean age was 34.4±9.99 years. Amongst them, 52% (43) were women with a mean age of 35.2±12.9 years, and 94.8% (786) were men with a mean age of 34.4±9.79 years.

Amongst the patients, 43.8% (357) had a positive malaria diagnosis. Amongst these patients, 40.6% (145) were infected with *P. falciparum*, 42.8% (153) with *P. ovale* and 0.8% [3] with *P. malariae*. Mixed infections of *P. falciparum* and *P. ovale* accounted for 6.1% [5], and mixed infections of *P. falciparum* and *P. malariae* accounted for 0.28% [1]; we will not include this one in our analysis, as it is not representative. A total of 9.2% (33) of patients could not be correctly identified due to too low parasitaemia. The chi-square test revealed a statistically significant difference between malaria diagnosis and gender (*P*-value=0.039). However, there was no statistically significant difference between gender and the *Plasmodium* species responsible for the infection (*P*-value=0.225).

The Shapiro–Wilk test indicated an abnormal distribution of our haematological parameters. Given the abnormal distribution of the data, we used the non-parametric Kruskal–Wallis test. There was no statistically significant difference between age and diagnosis (*P*-value=0.533). Using the Kruskal–Wallis test, we compared the median values of the previously mentioned haematological parameters and malaria diagnosis. The median values of platelets, Hb, white blood cells and lymphocytes in positive patients were significantly lower than those in negative patients (Table 1). The median MON in the positive group was greater than that in the negative group, with significant *P*-values of 0.048 and 0.003, respectively. There was no statistically significant difference between groups regarding NEU.

**Table 1.** Kruskal–Wallis test according to malaria status

	Malaria	PLT (10^3^ µl^−1^)	Hb (g dl^−1^)	LEU (10^3^ µl^−1^)	NEU (10^3^ µl^−1^)	LYM (10^3^ µl^−1^)	MON (10^3^ µl^−1^)
Mean	Negative	212±71.4	14.6±1.66	7.72±3.57	4.71±3.22	2.11±1.24	0.711±0.463
	Positive	117±57	13.7±1.75	6.4±2.56	4.41±2.34	1.2±0.862	0.719±0.34
Median	Negative	204	14.8	6.8	3.8	2	0.6
	Positive	112	14	6	4	1	0.7
*P*-value (Kruskal–Wallis)		<0.001	<0.001	<0.001	0.645	<0.001	0.048

Hb, haemoglobin; LEU, Leukocytes; LYM, lymphocyte count; MON, monocyte count; NEU, neutrophil count; PLT, platelet count.

**Fig. 1.**
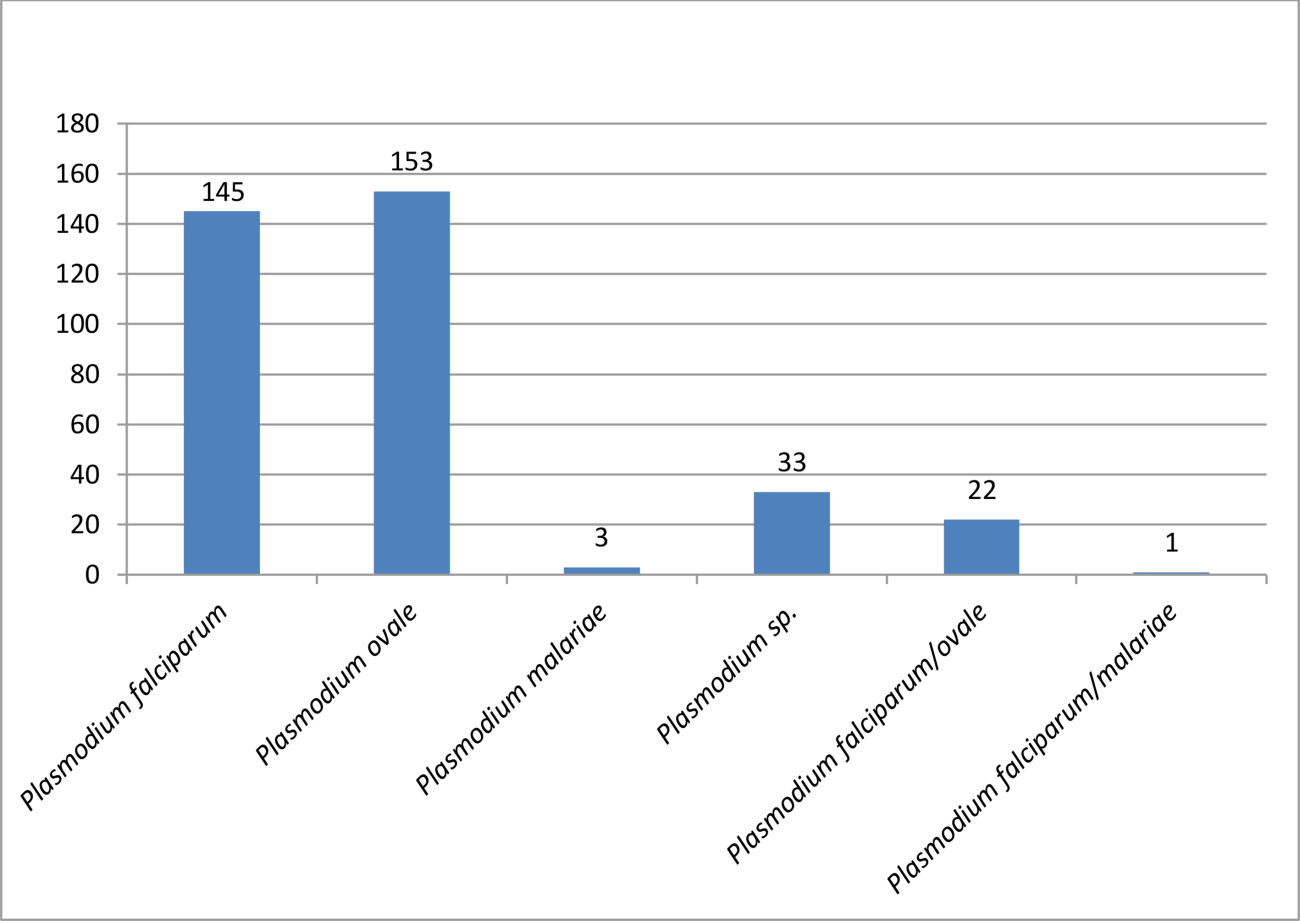
Case distribution according to species involved.

The mean parasitaemia in our series was 0.818±1.686, ranging from a minimum of 0.001%–18%. A total of 70% of positive patients (250) had parasitaemia below 1%, most of which were due to *P. ovale* (33.1%) (Fig. 1). A total of 24.9% (89) had parasitaemia between 1% and 4%, primarily due to *P. falciparum*. The majority of severe parasitaemia cases above 4% were due to *P. falciparum* (4.8%), with only one patient co-infected with *P. falciparum*/*P. ovale* also having parasitaemia above 4%.

An Hb level below 13 g dl^−1^ defines anaemia in men and below 12 g dl^−1^ in women. The median Hb level of the infected patients (13.7 g dl^−1^) was lower than that of the negative patients (14.6 g dl^−1^) (*P*-value<0.001) but remained above the anaemia threshold (Table 1). Eighty-nine patients positive for malaria were anaemic (65.4%), and 47 patients negative for malaria were also anaemic (34.6%) (Table 2). Using the anaemia definition threshold, we found a significant association between Hb level and malaria diagnosis (*P*-value<0.001). Hb level was inversely correlated with parasitaemia and malaria diagnosis (r=−0.295, r=−0.281, *P*-value<0.001).

**Table 2.** Diagnostic values of haematological parameters using Fisher’s exact test

Variable	Sensitivity	Specificity	PPV	NPV	OR	PLR	NLR	***P*-value**
Leucopenia (<4×10^3^ µl^−1^)	19.05%	72.25%	34.85%	53.38%	0.612483	0.686296	1.120514	0.003
Leucocytosis (>10×10^3^ µl^−1^)	8.12%	79.03%	23.19%	52.46%	0.333118	0.38729	1.162623	<0.001
Thrombocytopenia (<150×10^3^ µl^−1^)	75.91%	84.11%	78.83%	81.75%	16.68016	4.777292	0.286406	<0.001
Lymphocytopenia (<1.5×10^3^ µl^−1^)	69.47%	78.39%	71.47%	76.71%	8.253283	3.214588	0.389492	<0.001
Monocytosis (>1.1×10^3^ µl^−1^)	14.29%	86.65%	45.48%	56.47%	1.082011	1.070295	0.989172	0.698
Anaemia	24.93%	90.04%	66.12%	60.61%	3.002937	2.503606	0.833719	<0.001

NLR, negative likelihood ratio; NPV, negative predictive value; OR, odds ratio; PLR, positive likelihood ratio; PPV, positive predictive value.

Leucopenia is defined as a white blood cell count less than 4,000 µl^−1^. There was a significant difference in white blood cells (*P*-value<0.001) between the two groups. There were 34.2% (68) positive patients who were leucopenic versus 65.8% (131) negative patients who were leucopenic (*P*-value=0.004) (Table 2). The white blood cell count was inversely correlated with malaria diagnosis and parasitaemia (r=−0.19, r=−0.172, *P*-value<0.001). Leucocytosis is defined by a white blood cell count above 10*10^3^ µl^−1^. There were significant differences between the two groups for this abnormality according to Fisher’s exact test (*P*-value<0.001). A total of 8.1% (29) of the patients whose white blood cell count was above the cut-off value were positive for malaria, whilst 21% (99) of patients negative for malaria were such. The NEU did not significantly differ between the two groups (*P*-value=0.075) (Table 1). MON significantly differed between the two groups (*P*-value=0.048) (Table 1). The MON was correlated with malaria diagnosis (r=0.069, *P*-value=0.048) but was not correlated with parasitaemia. When we used the cut-off point (1.1×10^3^ µl^−1^) to define two groups (monocytosis and normal MON), we did not find a significant difference between them using Fisher’s exact test (*P*-value=0.908).

Lymphopenia is defined as an LYM below 1.5×10³ µl^−1^. The median of the positive malaria group [1] was lower than the median of the control group [2] with a *P*-value<0.001. A total of 69.5% (248) of patients positive for malaria had lymphopenia, whereas 21.6% (102) of patients negative for malaria had lymphopenia (Table 2). The LYM was correlated with diagnosis (r=−0.510, *P*-value<0.001) and parasitaemia (−0.216, *P*-value<0.001).

Thrombocytopenia is defined as a PLT less than 150×10³ µl^−1^. The median of the positive group (112) was lower than the median of the negative group (204), with a *P*-value<0.001 (Table 1). A total of 78.3% (271) of patients positive for malaria had thrombocytopenia, whilst only 21.7% (75) of the negative patients had thrombocytopenia (Table 3). Thrombocytopenia was correlated with diagnosis (r=−0.629, *P*-value<0.001) and parasitaemia (r=−0.649, *P*-value<0.001).

**Table 3.** Distribution of the severity of parasitaemia according to the species involved

		Parasitaemia (%)			
**Species**		**<1**	**1–4**	**>4**	**Total**
*P. falciparum*	Observed	83	45	17	145
	%	23.2	12.6	4.8	40.6
*P. ovale*	Observed	118	35	0	153
	%	33.1	9.8	0.0	42.9
*P. malariae*	Observed	3	0	0	3
	%	0.8	0.0	0.0	0.8
*Plasmodium* sp.	Observed	33	0	0	33
	%	9.2	0.0	0.0	9.2
*P. falciparum*/*ovale*	Observed	13	8	1	22
	%	3.6	2.2	0.3	6.2
Total	Observed	250	89	18	357
	%	70.0	24.9	5.0	100.0

### Diagnostic values of haematological parameters

We first performed a Fisher’s exact test to assess the odds ratio (OR), with a 95% confidence interval. Then, we calculated the sensitivity, specificity, positive predictive value (PPV), negative predictive value (NPV), positive likelihood ratio (PLR) and negative likelihood ratio (NLR). The results are presented in Table 2. Leucopenia’s sensitivity was 19.05%, specificity was 72.25%, PPV was 34.85% and NPV was 53.38%. The OR was 0.612, with a *P*-value of 0.003. Leucocytosis’s sensitivity was 8.12%, specificity was 79.03%, PPV was 23.19% and NPV was 52.46%. The OR was 0.333, the PLR was 0.387 and the NLR was 1.163, with a *P*-value of <0.001. Thrombocytopenia demonstrated a sensitivity of 75.91%, specificity of 84.11%, PPV of 78.83% and NPV of 81.75%. The OR was 16.680, with a *P*-value of <0.001. For lymphocytopenia, the sensitivity was 69.47%, specificity was 78.39%, PPV was 71.47% and NPV was 76.71%. The OR was 8.25, with a *P*-value of <0.001. Monocytosis had a sensitivity of 14.29%, specificity of 86.65%, PPV of 45.48% and NPV of 56.47%. The OR was 1.082, with a *P*-value of 0.698. Finally, anaemia showed a sensitivity of 24.93%, specificity of 90.04%, PPV of 66.12% and NPV of 60.61%. The OR was 3.003, with a *P*-value of <0.001.

### Combined variable analysis and determination of the optimal threshold for malaria diagnosis

A binomial logistic regression was used to predict malaria diagnosis, incorporating three independent variables: platelet count (PLT), Hb, and LYM. The regression coefficients were as follows: −0.0208 for PLT, −0.1859 for Hb and −0.8143 for LYM, with a constant value of 7.0013 (Table 4). The new predictive variable for each patient is expressed as: new malaria predictive variable=((−0.0208) PLT+(−0.1859) Hb+(−0.8143) LYM+7.0013)10.

**Table 4.** Logistic regression coefficients

Predictor	Regression coefficient	Standard error	***P*-value**
Intercept	7.0013	0.89063	<0.001
Platelets	−0.0208	0.00186	<0.001
Hb (g dl^−1^)	−0.1859	0.06049	0.002
Lymphocyte	−0.8143	0.12994	<0.001

We obtained a precision of 83.2%, a specificity of 86.9%, a sensitivity of 78.4%, a PPV of 81.87% and an NPV of 84.1%. The OR was 28.29, the PLR was 7.27, the NLR was 0.257 and the area under the curve (AUC) was 0.889 (Table 5).

**Table 5.** Diagnostic performance of the regression model

Precision	Specificity	Sensitivity	PPV	NPV	OR	PLR	NLR	Area under the curve
0.832	86.9%	78.4%	81.87%	84.1%	28.29	7.27	0.257	0.889

NLR, negative likelihood ratio; NPV, negative predictive value; OR, odds ratio; PLR, positive likelihood ratio; PPV, positive predictive value.

**Fig. 2.**
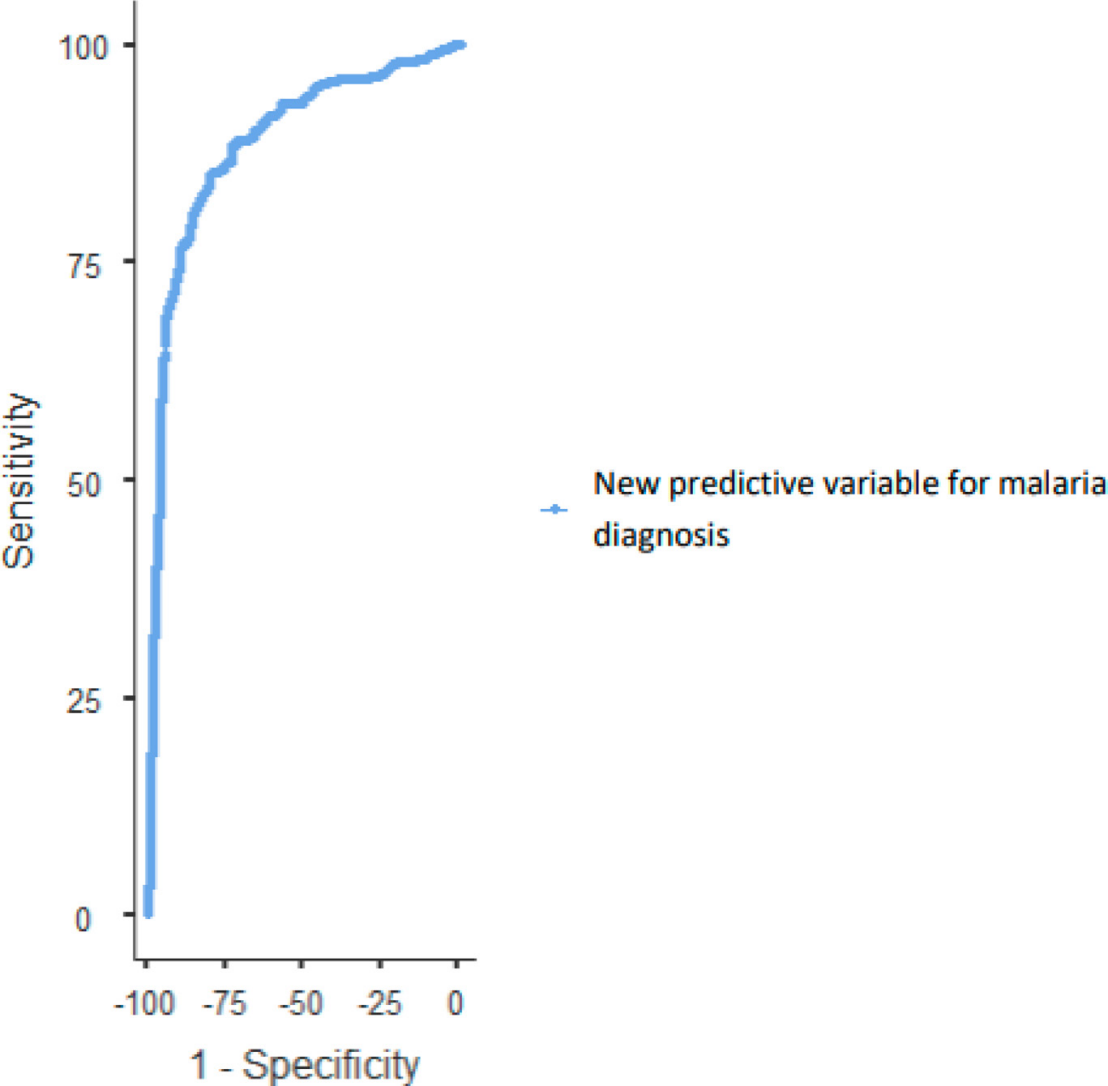
ROC curve of the new predictive variable for malaria diagnosis.

ROC curve analysis revealed an AUC of 0.889 (Fig. 2). The optimal cut-off value, determined via Youden’s index, was 1.4464 (Table 6). The model’s performance characteristics are indicated in this table.

**Table 6.** ROC curve of the regression model

Cut point	Sensitivity (%)	Specificity (%)	PPV (%)	NPV (%)	Youden’s index	AUC
1.4664	77.03	89.41	84.62	83.73	0.664	0.889

AUC, area under the curve; NPV, negative predictive value; PPV, positive predictive value.

## Discussion

The present retrospective and comparative study investigated the haematological abnormalities associated with malaria in patients returning from endemic areas. The primary objective was to identify the most relevant haematological parameters for malaria diagnosis on the basis of an in-depth analysis of data collected over 6 years. Amongst the haematological parameters tested, only total leucocytes and lymphocytes were significant in diagnosing malaria. Amongst the white cell lineage, platelet and Hb were also found as such.

As indicated in Table 1, the median total white blood cell count was significantly lower in the positive group (6×10^3^ µl^−1^) than in the control group (6.8×10^3^ µ^−1^), which is consistent with the findings of the study by Awoke and Arota [3]. The mean of the positive group (5.3±2.2) was lower than that of the negative group (5.8±0.8), as was the case in the study by Kotepui *et al*. [2] where the median leucocyte count of the *P. falciparum* group (5.76×10^3^ µl^−1^) was lower than that of the negative group (8.71×10^3^ µl^−1^). In our series, 19.04% (68) of the infected patients had leucopenia, whereas 8% (29) of the patients in the same group had leucocytosis. The predominance of leucopenia in our series is consistent with other studies, notably the study by Rasheed *et al*. [6], which reported leucopenia in 20.9% of *P. falciparum* cases. Similarly, 22.1% (32) of *P. falciparum* cases were leucopenic in our series. There was no statistically significant difference between the NEUs of the two groups, as confirmed by the study of Nfor Omarine Nlinwe and and meta analysis including 11 scientific articles on haematological abnormalities in malaria by Kotepui *et al* [7,8]. This finding contrasts with several studies that reported higher NEUs in the positive group compared to the negative group [3,9].

In our study, monocytosis (MON>1.1×10³ µl^−1^) was observed in a smaller proportion of malaria-positive patients than other haematological anomalies. Specifically, 14.3% (51) of the positive group exhibited monocytosis, whereas 86.7% (405) of the negative group did not. This difference was statistically significant, with a *P*-value of 0.048. The median MON in the positive group (0.719×10^3^ µl^−1^) was slightly greater than in the negative group (0.711×10^3^ µl^−1^), but the difference was not as pronounced as that in the other parameters (Table 1).

Our findings align with those of the study by Awoke and Arota [3], which reported an increase in MONs amongst malaria patients (Table 1). Chandra S. and Chandra H. reported that the median MON in the positive group was lower than that in the control group [10]. However, their study did not find it to be as prevalent as other haematological changes such as lymphopenia and thrombocytopenia. The slight increase in MON in malaria patients can be attributed to the body’s immune response to infection, where monocytes play a critical role in phagocytosis and cytokine production, which are essential for controlling parasitic infection [3]. Comparatively, Kotepui *et al*. [2] reported that the increase in MONs amongst malaria patients was not as significant as the reduction in other cell lines, such as lymphocytes and platelets. These authors suggested that whilst monocytosis can be a marker for malaria, it is less specific and sensitive than other haematological abnormalities are. This finding is consistent with our observation, where, compared with lymphopenia, monocytosis had a lower sensitivity (14.29%) and specificity (86.65%) for malaria diagnosis (Table 2). A study by Kini and Chandrashekhar [9] also revealed that MONs can be elevated in malaria patients. However, it emphasized that this change is often overshadowed by more significant alterations in other leucocyte subsets. They highlighted the variability in the monocyte response, which can be influenced by factors such as the severity of the infection, patient immune status and co-infections, leading to inconsistent findings across different studies. Monocytosis in malaria is likely part of a broader, complex immune response rather than a primary diagnostic marker. The immune activation leading to monocytosis might involve the release of cytokines and chemokines that recruit and activate monocytes at the site of infection. However, the extent and significance of monocytosis can differ, and it is influenced by the dynamic interactions between the parasite and the host immune system [11,12].

In our study, we found that the median LYM was significantly lower in the positive group (1×10^3^ µl^−1^) than in the negative group (2×10^3^ µl^−1^), as demonstrated by the meta-analysis conducted by Kotepui *et al*. [7]. Lymphopenia (LYM<1.5×10^3^ µl^−1^) was the most significant leucocyte abnormality observed in malaria patients, with 69.5% (248) of positive patients being lymphopenic, whereas 21.6% (102) of negative patients were such. Our results seem to align with those of the study by Awoke and Arota [3], which reported lymphopenia in 56.8% of positive patients. In another study, 20.1% of positive patients were lymphopenic, whereas only 4.6% of negative patients were such. This discrepancy with our study can be explained by the chosen lymphopenia threshold, which was 1.5×10^3^ µl^−1^ in our study versus 0.8×10^3^ µl^−1^ in the study by Kotepui *et al*. [2]. The cause of lymphopenia in malaria is likely not singular but multifaceted, with the leading causes differing from one another, as malaria infection induces a wide variety of host responses in each host-parasite system. This view is consistent with the fact that studies on malaria-associated lymphopenia have shown variable results, leading to different interpretations of this phenomenon. These combined mechanisms highlight the complex interaction between the parasite and the host immune response. We further speculate that the main cause of lymphopenia changes during the course of infection, even in the same host. In the early phase of the disease, lymphopenia is caused mainly by the redistribution of activated T cells. However, with disease progression, Fas-mediated apoptosis plays an increasingly important role in lymphopenia. This hypothesis aligns with observations that the degree of lymphopenia correlates with disease severity and that apoptotic cells are, as mentioned by Hviid and Kemp [12], difficult to detect except in moribund patients.

Anaemia is, according to the literature, one of the most common abnormalities in malaria, especially in young children and pregnant women [13]. The pathogenesis of anaemia during malaria infection is multifactorial and remains incompletely understood. It results from a combination of haemolytic mechanisms and accelerated removal of parasitized and non-parasitized red blood cells, suppression of erythropoiesis and bone marrow dysfunction [14]. The erythrocytic schizogony phase causes haemolysis, which is partly responsible for progressive anaemia, which is particularly severe in young children and pregnant women. The Hb released by this haemolysis causes renal overload and is partly converted into bilirubin in the liver, with the excess being excreted in the urine, leading to haemoglobinuria. The use of Hb by the parasite for its development results in the precipitation of pigment granules (hemozoin) in its cytoplasm, which are composed of haem that is toxic to the parasite and coated with parasitic proteins to render it inactive. The release of these granules during red blood cell rupture partly contributes to the observed fever. Abnormally high levels of TNF [14] and IL-10 [15] have also been associated with *P. falciparum* malaria anaemia due to their suppressive effect on the bone marrow. These cytokines cause bone marrow suppression and imbalance in red blood cell surface markers such as CR1 [16], contributing to anaemia. The phagocytosis of parasite-infected red blood cells is another mechanism involved in red blood cell destruction and worsening of anaemia. Our study reported a significant reduction in Hb levels in positive patients compared with negative patients (14 g dl^−1^ vs. 14.8 g dl^−1^, *P*-value<0.001).

These results are consistent with those of Kotepui *et al*. [2] study, which found significantly lower Hb levels in the infected group than in the healthy group. Similar results were reported in the study by Omarine Nlinwe and Nange [8]. The rate of severe anaemia (<5 g dl^−1^) in the malaria population in our study was 0.3% [1], whilst no severe anaemia cases were found in the control population. The Hb genotype profile of our particular region partly explains this protection from severe anaemia cases. Indeed, in the Mediterranean region, thalassaemia and sickle cell disease are more common than in other parts of the world. These haemoglobinopathies protect against severe forms of malaria [17,18]. According to a study by Alaoui *et al*. [19], the prevalence of haemoglobinopathies in Morocco has yet to be accurately estimated. However, Morocco is a high-prevalence country where the predominant haemoglobinopathy is sickle cell disease.

Additionally, our study was conducted at a military institution where most of our patients were military personnel educated in malaria management. Therefore, at the slightest symptom indicative of malaria, they seek emergency care where procedural management exists. Thus, early management is implemented, and severe cases are rare.

In our study, it was the most common abnormality during infection. Indeed, 75.9% (271) of the positive patients had thrombocytopenia (PLT<150×10^3^ µl^−1^), whereas only 15.9% (75) of the negative patients had thrombocytopenia. Our results are consistent with the studies by Awoke and Arota [3], which reported a prevalence of thrombocytopenia in 84% of infected patients, and the study by Kotepui *et al*., which found a prevalence of 84.9% [2], and the study by Gebreweld *et al*., which reported a prevalence of 79.5% [20]. Thrombocytopenia in malaria is a multifactorial complication involving several distinct mechanisms. Babker [21] suggested that it could be an indicator of malaria, although the precise pathways remain unclear. de Mast *et al*. [22] associated early thrombocytopenia in malaria with loss of glycoprotein-1b, mediated by von Willebrand factor, without systemic platelet activation or consumptive coagulopathy. In contrast, Witzemann *et al*. [5] identified pre-activation of platelets during early infections, involving platelet clearance mechanisms rather than activation. Lacerda *et al*. [23] provided a broader perspective, describing potential mechanisms such as coagulation disturbances, splenomegaly, bone marrow suppression and antibody-mediated platelet destruction. Finally, Santos *et al*. [24] highlighted the role of inflammatory mediators such as IL-10, IL-8/Hepatocyte growth factor (HGF) and miRNAs, suggesting that immune responses significantly contribute to severe thrombocytopenia in *P. vivax* infections. These studies illustrate that thrombocytopenia in malaria patients results from complex interaction amongst immune responses, platelet clearance mechanisms and specific molecular interactions.

In a subsequent analysis, we aimed to determine threshold values for haematological parameters to aid in the diagnosis of malaria, given their rapid availability in a blood test.

We initially chose an arbitrary threshold value for thrombocytopenia of 150×10³ µl^−1^ and performed Fisher’s test (Table 2). This threshold value has excellent sensitivity (75.91%) and specificity (84.11%), making it the best discriminating parameter in our study. These results are consistent with those of other studies, such as the study by Kotepui *et al*. [2], which reported a sensitivity and specificity of 85% with the same threshold value, and the study by Gebreweld *et al*. [20], which also reported a sensitivity of 79.5% and a specificity of 86.3%. However, we found positive and negative predictive values of 78.83% and 81.75%, respectively, which were not observed in the abovementioned studies. Gebreweld *et al*. [20] reported an NPV of 95.3%, whilst the PPV was much lower than our results (54.7%). Similarly, Kotepui *et al*. [2] found an NPV of 97%, whereas the PPV was 48%. Our exclusion criteria may explain this discrepancy, as we systematically excluded patients with conditions that induce thrombocytopenia. Thrombocytopenic patients were 16.68 times more likely to be infected with malaria than those without (OR=16.68), whereas Kotepui *et al*. reported an OR of 31.8 [2].

The chosen threshold for lymphocytopenia was <1.5×10³ µl^−1^. We obtained a sensitivity of 69.74% and a specificity of 78.39%. Other studies have chosen lower thresholds, such as the study by [25], which, with a lymphocyte threshold of 0.7×10³ µl^−1^, obtained a sensitivity of 33% and a specificity of 72%. In the study by Kotepui *et al*. [2], with a chosen threshold of 0.8×10³ µl^−1^, they obtained a sensitivity and specificity of 25% and 78%, respectively. Our PPV was much greater than those reported in the literature. For example, Kotepui *et al*. [2] reported a PPV of 17%. The same results were found by van Wolfswinkel *et al*., whose LYM below 0.7×10^3^ µl showed a PPV of 16%, whereas our NPV was lower [76.71% vs 86% (2) vs 87%] [25]. In our series, a patient with an LYM below 1.5×10³ µl^−1^ was 8.25 times more likely to be infected with malaria (OR: 8.2533), whereas Kotepui *et al*. reported a much lower OR (1.4) [2].

For the Hb parameter, we used the WHO thresholds on the basis of gender. For men, Hb must be below 12 g dl^−1^; for women, this threshold is 11 g dl^−1^. We obtained sensitivity and specificity values of 24.93% and 90.04%, respectively. Kotepui *et al*. [2] reported this low sensitivity (35%) and high specificity (68%). In our series, anaemic patients were three times more likely to be positive for malaria than non-anaemic patients, whereas Kotepui *et al*. reported an OR of only 1.2 [2]. Our PPV was higher than that of the Kotepui *et al*. study [2], with a PPV of 15%, whilst their NPV was higher, 88%, than that of our study (60.61%). In the study by Chandra S. and Chandra H., a higher PPV than ours was reported (76.2%) [10]. Our PPV results align with those of Kotepui *et al*. [2].

Leucopenia (<4×10³ µl^−1^) had a sensitivity of 19.05% and a specificity of 72.25%, which is consistent with the findings of the study by Kotepui *et al*. [2], which reported a sensitivity and specificity of 17% and 94%, respectively. A study by Chandra S. and Chandra H. confirmed our results with a sensitivity and specificity of 11.3% and 90%, respectively [10]. Whilst our PPV was mediocre (34.85%), the study by Chandra S. and Chandra H. [10] showed an excellent PPV of 79.2%.

Leucocytosis (>10×10³ µl^−1^) had a sensitivity and specificity of 8.12% and 79.03%, respectively, which was confirmed by Chandra S. and Chandra H. [10], who reported a sensitivity and specificity of 18.8% and 56%, respectively, for a leucocyte count above 11×10³ µl^−1^. For leucopenia, our PPV was lower than that reported by Chandra S. and Chandra H. (58.9%) [10]. These results suggest that leucopenia has a limited diagnostic utility because of its poor sensitivity and moderate specificity; the low OR (0.612) indicates a weak association between malaria and leucopenia. The same interpretation applies to leucocytosis.

Whilst some parameters showed excellent diagnostic performance, such as PLT and LYM, Hb and total leucocyte count showed moderate diagnostic performances. The use of a combination of parameters is consistent, as these parameters are obtained simultaneously in the same blood analysis. We combined PLT, LYM and Hb level in a binomial logistic regression. Our results are compared to those obtained by Paintsil *et al.* [26] in their study on haematological predictors of malaria infection in a region of Ghana.

The regression coefficients for age, Hb, platelets and lymphocytes in the study by Paintsil *et al*. [26] were −0.041, −0.183, −0.011 and −0.031, respectively. Our results revealed a coefficient of −0.0208 for platelets, −0.1859 for Hb and −0.8143 for lymphocytes. The negative coefficients in both studies suggest that higher levels of these parameters are associated with a reduced likelihood of malaria infection.

Paintsil *et al*. reported a sensitivity of 77.4% and a specificity of 75.7%. Our model had a sensitivity of 78.4% and a specificity of 86.9%, which are higher than those of their model. High sensitivity is crucial for minimizing false negatives, whereas high specificity helps reduce false positives.

The PPV and NPV in the study by Paintsil were 52.72% and 90.51%, respectively. Our values were 81.87% and 84.1%, indicating that our model has a higher PPV.

In the study by Paintsil, the AUCs for platelets, Hb, lymphocytes and age were 0.772, 0.650, 0.605 and 0.600, respectively. Our ROC analyses revealed a combined AUC of 0.889 for our new predictive value, which contrasts with their results. An AUC greater than 0.7 is generally considered indicative of good diagnostic performance. Additionally, we determined a threshold value usable in laboratory settings to facilitate diagnosis, with a Youden’s index of 0.664, indicating good diagnostic performance of the calculated threshold value of 1.4664, with a sensitivity of 77.03% and a specificity of 89.41%. Thus, the results are comparable to those of Paintsil *et al*. [26]. The obtained PPV was higher than that of the model by Paintsil *et al*. [26]

Amongst the limitations of this study is the partial absence of information on patient comorbidities and histories, such as the presence of haemoglobinopathies or infections other than malaria, which could influence the trends in haematological parameters. Additionally, at our institution, most of our patients were military personnel educated on malaria management, which may influence our results.

## Conclusion

This study comprehensively analysed the haematological abnormalities associated with malaria in patients returning from endemic areas. Our findings indicate that haematological parameters such as PLT, Hb level and LYM are significant indicators for malaria diagnosis.

In conclusion, our study reinforces the importance of specific haematological parameters in diagnosing malaria and suggests that a combined diagnostic approach can significantly improve accuracy. These findings have practical implications for the early and accurate diagnosis of malaria, particularly in travellers returning from endemic regions, and can aid in initiating appropriate treatment. Further research is recommended to explore the potential of these haematological markers in different populations and clinical settings.

## Supplementary material

10.1099/acmi.0.000919.v3 Supplementary Material 1.

## Abbreviations

AUC: area under the curve
Hb: haemoglobin
LEU: leukocytes
LYM: lymphocyte count
MON: monocyte count
NEU: neutrophil count
NLR: negative likelihood ratio
NPV: negative predictive value
OR: odds ratio
PLR: positive likelihood ratio
PLT: platelet count
PPV: positive predictive value
ROC: receiver operating characteristic
